# Youth as digital citizens in health: Experiences, challenges, and the road ahead

**DOI:** 10.1371/journal.pdig.0000923

**Published:** 2025-07-08

**Authors:** Aishwarya Rohatgi

**Affiliations:** Independent Researcher, New Delhi, India; Fundação Oswaldo Cruz: Fundacao Oswaldo Cruz, BRAZIL

Imagine a 20-year-old waking up to news of a new virus outbreak in their country, instantly delivered through a news app or social media platform. In the following days, as flu-like symptoms resembling the newly identified variant emerge, they turn to an ENT specialist for a teleconsultation. After receiving the prescription, they upload it to their personal health app, which uses GIS technology to map the nearest pharmacy. They select the most convenient one, place an order for the medication, and have it delivered to their home within minutes through a hyper-local delivery service.

This is the reality for most young people today. Growing up as digital natives, they live in a world where technology—smartphones, the internet, social media—shapes how they communicate, learn, and navigate daily life. When it comes to healthcare, they seek seamless, tech-driven solutions that are efficient, convenient, affordable, and secure. Their expectations should be central to the design of digital health interventions, ensuring these systems are accessible, trustworthy, and aligned with their needs. From wearables on their wrists to policies governing the protection of their personal health data, young people’s preferences must be considered.

Despite being key stakeholders, young voices are often excluded from decision-making processes, particularly in healthcare and digital transformation. This is largely due to the perception that they lack lived experience or technical expertise, which diminishes the value of the fresh perspectives they bring. Many young people feel sidelined, resulting in limited engagement and advocacy in shaping digital health solutions. This disconnect not only overlooks a generation that is inherently tech-savvy but also impedes the development of inclusive and forward-thinking healthcare innovations.

As primary users of healthcare services—often facilitated through digital tools—the experiences and insights of young people are essential in driving the digitization of health systems. They don’t just access care; they also contend with challenges such as health misinformation and online harms. Engaging youth as active participants, not just passive users, is crucial in building a digital health ecosystem that is responsive and proactive.

Understanding how public and private sectors can collaborate to empower youth in digital health is complex, but several concrete steps can be taken to ensure young people are not just recipients of digital health interventions but active contributors.

First, youth representation must be prioritized in policy discussions, research initiatives, and the design and deployment of digital health solutions. Their lived experiences and digital fluency can help ensure that digital health systems meet their needs, making them more effective. For instance, developing national digital health strategies should involve consultations with diverse youth groups. A targeted needs assessment capturing their perspectives will help shape policies and solutions for long-term adoption, while addressing concerns like health data privacy in accessible and transparent ways.

Second, young people must be equipped with both health and digital literacy to become change agents within their communities. Their ability to guide the elderly, support those with limited digital access, or advocate for informed health choices underscores their role in bridging health inequities through digital empowerment. This not only amplifies their voices but enables them to actively participate in creating communication materials and articles about the digital health landscape of their country.

Lastly, youth should be encouraged and supported in forming networks and advocacy groups to drive awareness and promote user-centric digital health solutions. Youth-led movements can influence the creation of safer, more responsive digital health systems that cater to all. A youth-centered digital health interventions framework can help plan, develop, and implement digital solutions that address their unique health challenges, benefiting both young people and the broader digital health community.

Several youth-led initiatives, like Young Experts: Tech for Health (YET4H), are already shaping discussions and advocating for youth leadership in digital health. It’s essential that these platforms ensure diverse representation across gender, socio-economic status, citizenship, and disability to uphold equity and inclusivity. Public-facing engagements across organizations should prioritize young people, placing them at the forefront of these conversations.

Academia and research institutions play a key role in empowering youth in digital health by ensuring their representation in research proposals, ethics committees, and implementation teams. These institutions can facilitate youth-centered digital tool design, conduct human-centered research, test innovations with young users, and refine best practices for usability and accessibility. Mentorship programs, research fellowships, and workshops in digital health, AI, and telehealth can also help equip young professionals with the skills to drive inclusive and scalable digital health solutions.

By taking these steps, we can ensure that youth are not merely beneficiaries of digital health but active architects of its future. Their blend of technological fluency, innovation, and lived experience makes them invaluable contributors to shaping the design, implementation, and advocacy of digital health solutions. To realize their full potential, a shift is needed—moving from passive recipients to active decision-makers in healthcare transformation.

Youth representation in policymaking, research, and digital tool development is not just an inclusion exercise—it is a necessity for building equitable, effective, and sustainable digital health systems. By investing in their education, amplifying their voices, and fostering youth-led advocacy, we can create a digital health ecosystem that responds to their needs and empowers them to lead future innovations. The path forward lies in collaboration—governments, academia, the private sector, and civil society must work together to integrate youth perspectives at every stage.

A truly inclusive digital health future is one where young people are not only participants but pioneers, shaping a world where technology enhances healthcare for all.

